# Bacteria Associated with Prostate Cancer Progression and New Strategy in the Treatment

**DOI:** 10.34172/apb.025.45862

**Published:** 2025-06-05

**Authors:** Fathimath Ina Shareef, Kannan Subbaram, Razana Faiz, Sheeza Ali

**Affiliations:** School of Medicine, The Maldives National University, Malé, Maldives

## To the Editor,

**Figure 1:**
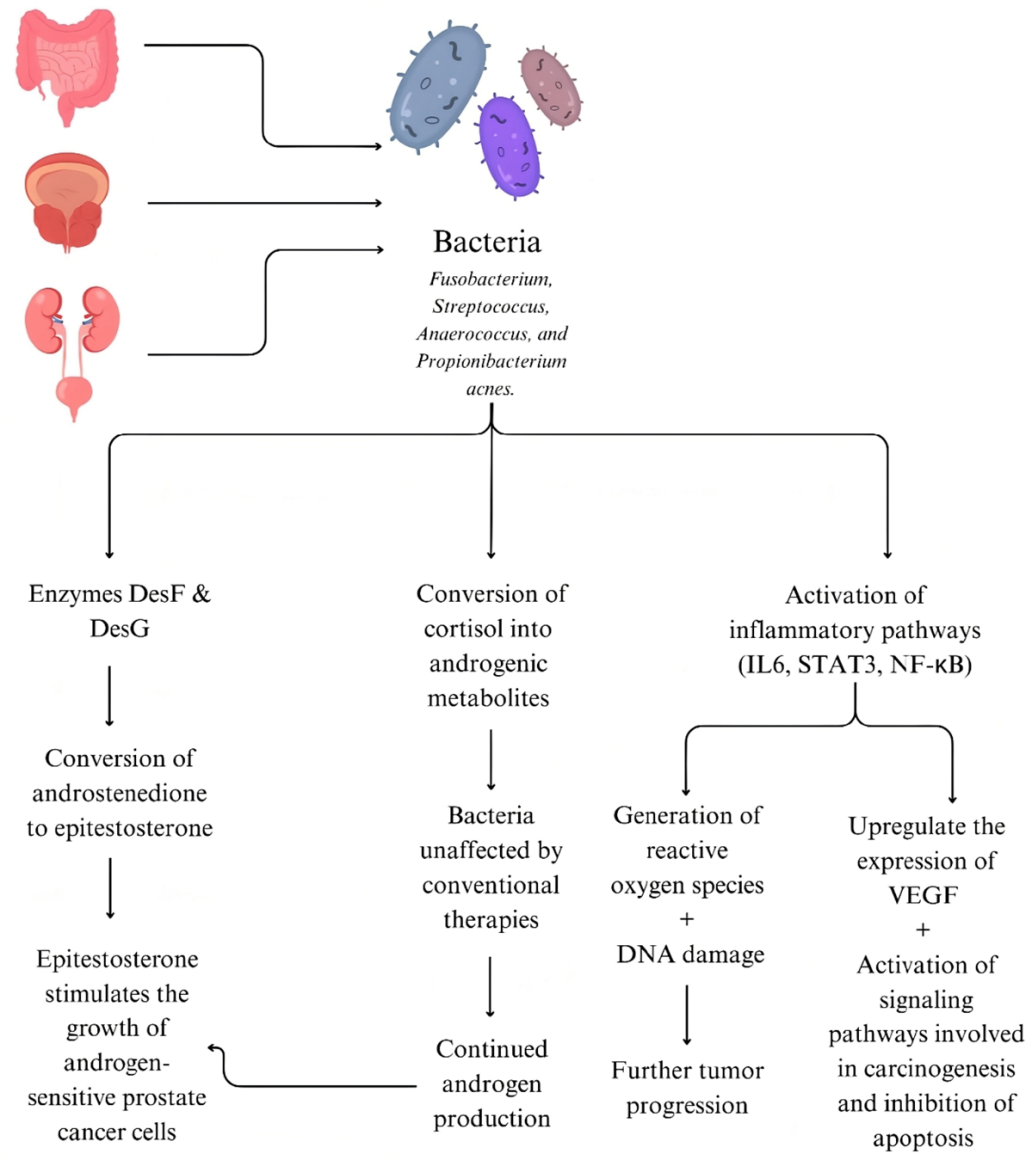
Association of bacteria in prostate cancer progression

 Globally, prostate cancer ranks as the second most prevalent malignancy affecting men. Although the incidence and mortality show a decreasing trend, it is still one of the leading causes of cancer related mortality. There are multiple factors which contribute to the development of prostate cancer. Established risk factors include age, ethnicity, family history and even environmental factors.^1^ Recent studies suggest that bacteria present in the gut, prostate tissue and the urinary tract may play a role in the development and progression of prostate cancer. Examples of such bacterial species include *Fusobacterium, Streptococcus, Anaerococcus*, and *Propionibacterium acnes*. Opportunistic pathogens such as *Actinomyces* species and *Varibaculum cambriense* are also elevated in cancer tissue.^2^ This finding raises questions about the mechanisms through which bacteria may influence prostate cancer development and progression. One proposed mechanism may be due to the enzymes desmolase F (DesF) and desmolase G (DesG), which convert androstenedione into epitestosterone. These enzymes were isolated from *Clostridium scindens* and *Propionimicrobium lymphophilum* species (Figure 1). Epitestosterone stimulates the growth of androgen-sensitive prostate cancer cells, which is even more sustained than the effects of testosterone.^3^ Another mechanism includes bacteria which are capable of converting cortisol into androgenic metabolites. These species were found more commonly in the urine samples of patients who went on to develop prostate cancer. These bacteria remained unaffected by conventional therapies, allowing the androgen production to continue despite androgen-deprivation therapy.^4^ Another theory suggests the induction of a chronic inflammatory state by some bacterial species, which promotes oncogenesis. Inflammatory mediators upregulate the expression of vascular endothelial growth factor and activate multiple signaling pathways involved in carcinogenesis and inhibition of apoptosis.^4^ A persistent inflammatory environment promotes tumor formation. Presence of some bacterial flora such as *Proteobacteria* activated inflammatory pathways such as IL6, STAT3, and NF-κB. These pathways generate reactive oxygen species and cause DNA damage, further promoting tumor progression.^2^ Moreover, *Propionibacterium acnes* inoculation in mice was found to cause severe acute and chronic inflammation of the prostate, with chronic inflammation persisting up to 8 weeks.^5^ These findings open up new avenues for therapeutic intervention in prostate cancer. Microbial components may even be used as biomarkers in prostate cancer. Currently, prostate specific antigen is the most widely used marker although it has notable limitations. Prostate cancer may occur even if prostate specific antigen is below the conventional diagnostic threshold of 4.0 ng/mL.^1^ MicroRNAs of urine microorganisms and human endogenous retrovirus sequence are associated with prostate cancer, making them possible promising alternatives.^1^ Despite advancements in conventional therapies, risks of residual disease and biochemical recurrence remain significant challenges.^1^ Addressing the pitfalls is vital in reducing these risks. Treatment adjuncts which target the implicated bacterial species may enhance the efficacy of current treatments, eventually reducing the morbidity and mortality associated with prostate cancer.

 Antibiotic therapy targeting the implicated bacteria is one possible adjunct to the conventional therapies. However, this approach must be exercised with caution, as there are risks of antibiotic resistance and disruption of commensal microbiota; leading to further complications. Use of prebiotics, fecal microbiota transplantation are other options worth exploring.^6^ New evidence showing the significant roles of microbiome in its development and progression opens new opportunities to improve current diagnostic and therapeutic strategies. Incorporating microbial components as biomarkers along with existing screening tools is one promising new strategy. Furthermore, targeted interventions against implicated bacterial species as an adjunct to current conventional therapy may help drastically improve treatment outcomes.

## Conclusion

 Prostate cancer in the second most prevalent cancer among men worldwide. Emerging evidence suggests association between certain bacterial species – such as *Fusobacterium, Streptococcus, Anaerococcus*, and *Propionibacterium acnes*– and prostate cancer. These microbes contribute to the development and progression of prostate cancer through several mechanisms, including increasing epitestosterone levels via bacterial enzymes DesF and DesG, and activation of several inflammatory pathways. These findings open up new avenues in diagnostics and therapeutic interventions, such as including antibiotics and fecal microbiota transplantation in addition to conventional therapies. Further research is vital in developing more precise, targeted therapies. Incorporating these strategies in clinical practice has the potential to markedly improve patient outcomes.

## Competing Interests

 The authors declare that there are no conflicts of interest.

## Ethical Approval

 Not applicable.
